# Perceptual Learning of Motion Leads to Faster Flicker Perception

**DOI:** 10.1371/journal.pone.0000028

**Published:** 2006-12-20

**Authors:** Aaron R. Seitz, Jose E. Nanez, Steve R. Holloway, Takeo Watanabe

**Affiliations:** 1 Department of Psychology, Boston University Boston, Massachusetts, United States of America; 2 Department of Social and Behavioural Science, Arizona State University Phoenix, Arizona, United States of America; Centre de Recherches su la Cognition Animale - Centre National de la Recherche Scientifique and Université Paul Sabatier, France

## Abstract

Critical flicker fusion thresholds (CFFT) describe when quick amplitude modulations of a light source become undetectable as the frequency of the modulation increases. The threshold at which CFF occurs has been shown to remain constant under repeated testing. Additionally, CFF thresholds are correlated with various measures of intelligence, and have been regarded by clinicians as a general measure of cortical processing capacity. For these reasons, CFF is used as a cognitive indicator in drug studies, as a measure of fatigue, and has been suggested as a diagnostic measure for various brain diseases. Here we report that CFFT increases dramatically in subjects who are trained with a motion-direction learning procedure. Control tasks demonstrate that CFFT changes are tightly coupled with improvements in discriminating the direction of motion stimuli, and are likely related to plasticity in low-level visual areas that are specialized to process motion signals. This plasticity is long-lasting and is retained for at least one year after training. Combined, these results show that CFFT relates to a specialized sensory process and bring into question that CFFT is a measure of high-level, or general, processes.

## Introduction

For more than two centuries, researchers have studied how quick amplitude modulations of a light source (i.e. flicker) become undetectable as the frequency of modulation increases [1]. This in fact is the principle underlying modern cinematography and television and computer displays. In scientific investigations, critical flicker fusion threshold(s) (CFFT) are defined as the lowest rate of continuous flicker that is perceived as a steady source of light. Although retinal [2] and thalamic [3] neurons respond to flicker at rates over 100 Hz, perceptual studies show flicker cannot be detected at frequencies nearly this high.

The mechanisms that underlie CFFT are a topic of great interest. In 1947, Halstead found significantly lower CFFT in patients with frontal lesions. He and others also found strong correlations between CFFT and various measures of intelligence [4]–[6]. This, combined with evidence that CFFT is stable to repeated testing [7]–[9], has been taken as evidence that CFFT is a consistent and general measure of cortical processing capacity. Based on these findings, CFFT is used in medical and drug studies as a diagnostic tool. For instance, CFFT has been suggested as a diagnostic measure for Schizophrenia [10], Alzheimer's Disease [11], Multiple Sclerosis [12], and also some ocular diseases [13], [14]. In addition, CFFT is used as a measure of cognitive side-effects in psychopharmacological studies [15]–[17] and as a measure of workplace fatigue [18].

While there is considerable evidence of the relationship between CFFT and cortical processing capacity, lesion studies in non-human primates indicate that processing in the magnocellular visual pathway [19], [20] and occipital lobe [6], [21] are rate limiting for CFFT. Additionally, preliminary evidence suggests that manipulating visual experience modulates CFFT [22]–[24], although the mechanisms for this are unclear. Thus while there is general agreement that CFFT is cortical in origin, most neuroscience research in animals points towards CFFT being largely mediated by cells in the magnocellular visual pathway, which are specialized to process high temporal frequencies, respond to low-luminance contrasts, and are involved in motion processing [25], [26].

**Figure 1:**
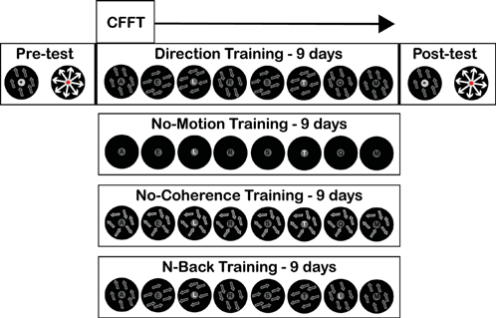
Experiment Design. For each group a direction discrimination test was performed before and after 9 days of training and CFFT was measured at the beginning of each training session. In Direction-Training, subjects reported two-targets (shown in white) at the end of the trial and a specific direction of motion was paired with task-targets. In No-Motion training, task was the same, but no dots were displayed. In No-Coherence training, task was the same but dots all moved randomly. In N-Back training, task was to report if a letter was repeated twice in a trial (in this case the L; shown in white for graphic purposes), there was no relationship between task-targets and motion directions.

Here we test the hypothesis that CFFT in humans is related to low-level motion processing by employing a visual training procedure that has been demonstrated to yield performance improvements specific to a particular direction of motion [27] and which are thought to arise from plasticity in low-level visual areas [28]. In this procedure (Figure 1), a sub-threshold motion-stimulus is temporally-paired with the targets of a letter identification task, and with many days of training on this task subjects develop improved sensitivity for the “paired-direction” when evaluated with tests of motion-direction discrimination [29].

## Results

**Figure 2:**
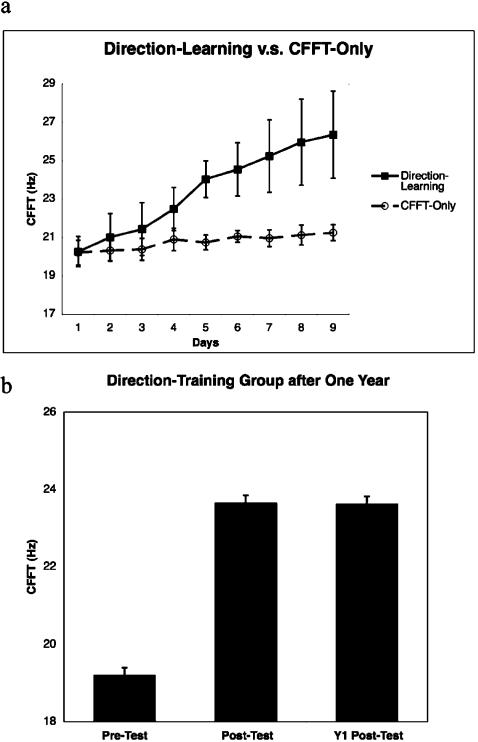
CFFT increases from Direction-Training. a, CFFT are shown for each day for the Direction-Training Group (solid-line) and the Flicker-Only Group (dashed-line). b, pre-training, post-training, and 1-year post-training results for subjects in the Direction-Training group who were re-tested 1-year after the conclusion of the training procedure. Error bars reflect standard-error.

We trained five subjects on this task (Direction-Training Group), and measured CFFT of subjects before they conducted each training-session on each of the nine-days of the training period. While we hypothesized a modest increase in CFFT would accompany improvements in discriminating coherent motion directions, we found to our surprise that thresholds increased quite substantially, on average by 30% (range 21–54%; see Figure 2a, solid-line) and showed no sign of reaching an asymptote by the end of training.

All (5 of 5) subjects in the Direction-Training Group showed a significant correlation of CFFT with days-of-training of at least p<0.01, with r^2^ ranging from 0.77–0.94. In comparison, five subjects in a control group (CFFT-Only Group), who did not conduct the training-task, showed only a 5% (range 3–9%) increase in CFFT across the nine-days of testing (see Figure 2a, dashed-line) and only 1 (of 5) subject showed significant correlation (at p<0.01) between training-day and CFFT. Comparing performance changes of the Direction-Training vs. CFFT-Only Groups showed a significant interaction (p<0.01; ANOVA) between day of training and group.

To test if repeated measures of CFFT were necessary for this effect we recruited three new subjects (PreTest-PostTest Group), who underwent the direction-training procedure, but had CFFT measured only twice; once before and once after the training-period. All of these subjects also showed robust and significant increases in CFFT (mean 22%; range 18–27%; p<0.01, t-test pretest vs. posttest).

To test if the elevations of CFFT in the Direction-Training Group were stable, we brought back 3 (of 5) subjects one-year after the initial study and again measured their CFFT. Remarkably, their post-training CFFT levels were highly stable and changed less than 1% in a year's time (see Figure 2b).

**Figure 3:**
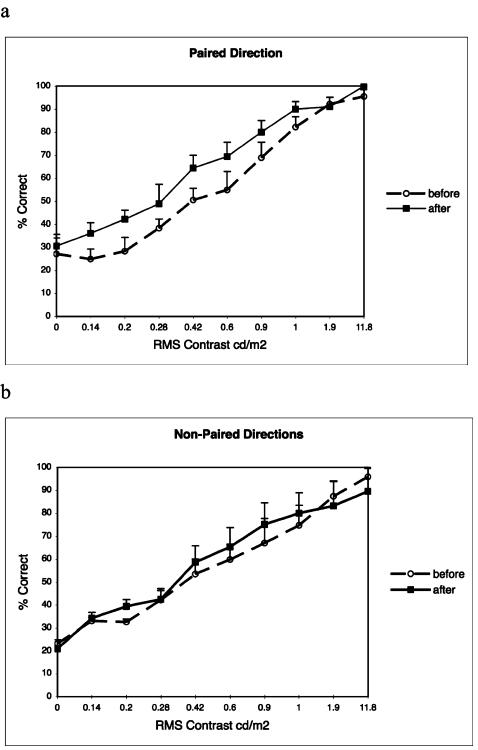
Motion-direction sensitivity change from Direction Training. a, performance for paired-direction on pre-test (dashed-line) and post-test (solid-line). b, performance for averaged across non-paired directions on pre-test (dashed-line) and post-test (solid-line). Error bars reflect standard-error.

For the Direction-Training Group we measured sensitivity for discriminating various motion directions before and after the training-period. Consistent with previous results [27], [29], improved performance was specific to the paired-direction. For this direction, which was paired with the task-targets, a significant improvement in performance was observed (p<0.01, ANOVA; Figure 3a) between the pre-test and post-test. No performance change was found for the non-paired directions (p = 0.34, ANOVA; Figure 3b). The fact that performance improvements in the direction task were specific to a particular direction of motion implies that the CFFT increase is not due to general improvements in cognitive, or even visual abilities, but is instead linked to the improvement of a highly specific visual skill.

To clarify what aspect of our training procedure led to these learning effects we ran a series of control studies in which we manipulated aspects of the tasks that subjects conducted and the motion-direction stimuli that were presented during the task. We reasoned that if the changes in CFFT were related to changes in motion-direction discrimination abilities then conditions that do not elicit improved motion-direction discrimination would yield no changes in CFFT.

**Figure 4:**
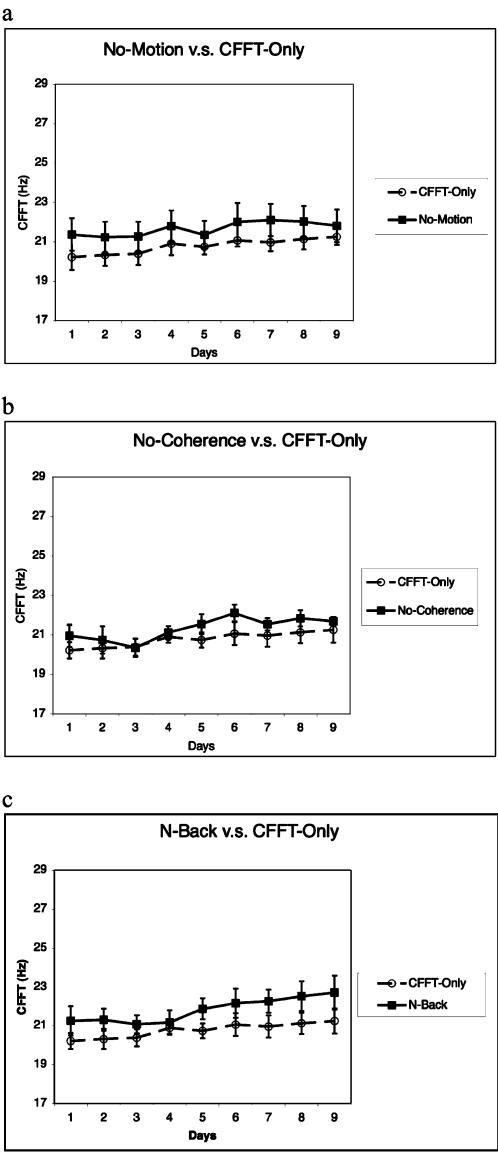
CFFT changes for control groups. a, CFFT is shown for each day for the No-Motion Group (solid-line) and the Flicker-Only Group (dashed-line). b, CFFT is shown for each day for the No-Coherence Group (solid-line) and the Flicker-Only Group (dashed-line). a, CFFT is shown for each day for the N-Back Group (solid-line) and the Flicker-Only Group (dashed-line). Error bars reflect standard-error.

We first asked if exposure to motion-direction stimuli (i.e. moving-dots) was required for CFFT to increase. Five new subjects were recruited (No-Motion Group) and trained with the same procedure that was used for the Direction-Training Group, with the exception that no moving-dots were presented during the training sessions. Learning would be expected for subjects if CFFT improvements were a consequence of the flickering of the letter stimuli used in the RSVP task, monitor refresh, or other such environmental factors. Contrary to this hypothesis, subjects showed on average only a 2% increase (range 0–6%) in CFFT across the nine-days of testing (see Figure 4a) and no subjects showed significant correlations between days-of-training and CFFT, or a significant interaction of group and training-day when compared with the Flicker-Only Group (p = 0.99; ANOVA).

We next asked if CFFT increases occurred merely due to the exposure with the flickering pattern of the moving-dots or if they were related to the training of motion-directions. Five new subjects were recruited (No-Coherence Group), who underwent the direction-training procedure, but moving-dots were presented at 0% coherence, instead of 100% coherence (used for Direction-Training Group), during the training sessions. If learning is due to the flickering of the moving-dots then CFFT increases should be expected from this task. Contrary to this hypothesis, subjects showed on average only a 4% improvement (range −5–13%) in CFFT across the nine-days of testing (see Figure 4b) and only 2 (of 5) subjects showed significant correlations between days-of-training and CFFT. There was no significant interaction of group and training-day when compared with the Flicker-Only Group (p = 0.97; ANOVA).

The data thus far indicate that exposure to coherently moving directional stimuli is necessary for significant CFFT increases to occur. Why do we find large changes in CFFT whereas many other groups have found CFFT to be remarkably stable? We have partially addressed this question by demonstrating that particular combinations of stimuli (i.e. coherent motion-direction paired with the letter task) are required for CFFT increases and that repeated CFFT testing, in the absence of the Direction-Training, yields very stable measurements. Another clue to the answer is found in our previous work, which shows that sensitivity-improvements of motion-direction stimuli requires a temporal-pairing between a motion-direction and a task-target [27], [29]. These results have led to a model of perceptual learning that shows how visual sensitivity improvements can occur through the coincidence of stimulus and reinforcement signals during training [30]. If this model is correct, and if CFFT increases accompany motion-direction sensitivity improvements, then manipulating the relationships between the motion-direction stimuli and the task-targets should affect whether CFFT changes.

To test this hypothesis we manipulated the training-task to disrupt the pairings between the task-targets and the motion-direction stimuli while preserving the visual presentation of the stimuli. Five new subjects were recruited (N-Back Group), who conducted a modified version of the direction-training procedure where an n-back task (see methods) was performed on the RSVP stimuli while 100% coherent motion-direction was presented in the periphery. In this task subjects were asked to report if the same letter appeared twice in a given trial, which occurred on 5% of the trials, and there was no systematic pairing between motion-direction and the n-back targets. Therefore, if the repeated pairing between the task targets and directional stimuli is required for CFFT increases, as they have been already shown for directional learning [27], then no CFFT change is expected from this task. On the other hand if CFFT changes merely from exposure to coherent motion-direction stimuli then CFFT increases should occur.

In accord with the target-pairing hypothesis we found no significant learning in this condition. For the N-Back group, CFFT showed on average a 7% increase (range 0–17%) in CFFT across the nine-days of testing (see Figure 4c) and only 1 (of 5) subject showed significant correlations between days-of-training and CFFT. There was no significant interaction of group and training-day when compared with the Flicker-Only Group (p = 0.87; ANOVA). We also failed to find consistent changes in CFFT in another group of subjects (N-Back-0% Group), who conducted the n-back task with 0% coherent moving dots (mean increases 5%; range 0–15%; with only 1 (of 4) subject showing correlation between days-of-training and CFFT). These results, combined with those of the previous experiments, show that both the strength of the stimulus signals and the pairing with target-related reinforcement signals are important for CFFT changes to occur.

**Figure 5:**
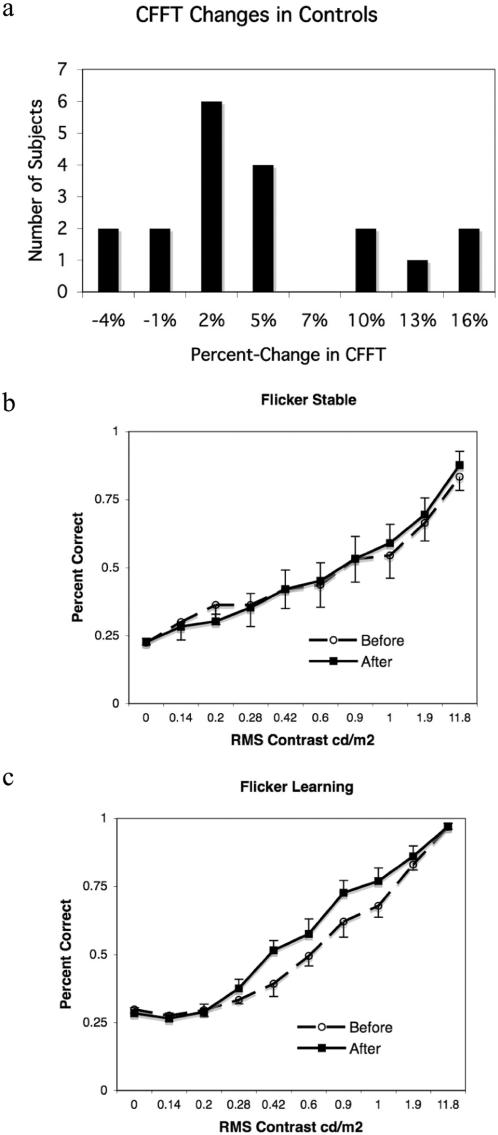
CFFT increases and motion-direction sensitivity for control groups. a, Histogram of percent-change in CFFT for subjects in the No-Motion, No-Coherence, N-Back and N-Back-0% Groups. b, performance averaged across directions for Flicker-Learning subjects on pre-test (dashed-line) and post-test (solid-line). b, performance averaged across directions for Flicker-Stable subjects on pre-test (dashed-line) and post-test (solid-line). Error bars reflect standard-error.

While we failed to find consistent CFFT increases in any group other than the Direction-Training Group, some individual subjects in the control groups (i.e. groups other than Direction-Training) did show changes in CFFT. This can be seen in Figure 5a, where a histogram of percent-changes in CFFT is presented for all the control groups. It is immediately clear that there is a bimodal distribution of CFFT changes, whereas most subjects (CFFT-Stable; 14 of 19) show 5% or lower, changes in CFFT, a second group of subjects (CFFT-Improving; 5 of 19) show 10–17% increases.

Given the hypothesized link between sensitivity-improvements for motion-direction stimuli and CFFT increases, we must examine how the motion-direction sensitivity of control subjects was affected by training. In line with our hypothesis, only individuals with elevated CFFT showed sensitivity-improvements for motion-directions. This can be seen in Figure 5b,c where psychometric functions from the direction task are plotted separately for the CFFT Stable and the CFFT Improving control subjects. While subjects in the CFFT Improving group showed a significant increase of performance on the direction task (p<0.05, ANOVA; Figure 5b), subjects in the CFFT Stable group showed no significant change in performance on the direction task (p = 0.64, ANOVA; Figure 5c).

## Discussion

Combined the results of these experiments seriously challenges the idea that CFFT is an immobile and general indicator of cortical processing. We have shown that CFFT can be significantly elevated through psychophysical training. These changes in CFFT co-occur with improvements of motion-direction sensitivity, which have previously been shown to result from plasticity in low-level visual areas [28]. In addition, this plasticity is long lasting and is retained for at least a year after the end of the training procedure.

The fact that CFFT is greatly elevated through a training procedure that results in sensitivity-improvements favoring a specific direction of motion brings into serious question the reliance on CFFT as a general measure of cortical processing or intelligence. Our results indicate that strengthening of a highly specialized visual skill (i.e. specific to a particular direction of motion), which presumably results from plasticity in a small subset of visual neurons, can result in CFFT elevations. While correlations with IQ across a large population may be real, such a relationship must be unreliable for individual patients whose CFFT could be altered in ways that are very specific to those patients' visual experience and abilities.

An important consideration in evaluating any study of CFFT is that different conditions of testing can produce very different thresholds. While, as we have shown, CFFT can be very stable to repeated testing, the exact thresholds observed are dependent upon luminance [31], eccentricity [32], spectrum of light [33], [34], size of light-source [35], state of adaptation to lighting conditions [36], etc.. These factors need to be taken into consideration when evaluating CFFT across studies or even across sessions within studies. In our case, all stimulus conditions relating to CFFT measurement were kept constant for all subjects in all sessions in all studies and this is evidenced by the fact that thresholds remained steady for most subjects.

Our results showing a relationship between processing of coherent motion-directions and that of CFFT are consistent with properties of cells in the magnocellular visual system, which are known to play a critical role in both these perceptions. Starting off in the retina, research indicates that responses of magnocellular ganglion cells (i.e. parasol cells) show minima of phasic activity that correspond well to heterochromatic flicker fusion thresholds of humans [34]. On the other hand, evidence that the LGN and V1 can respond to flicker at rates approaching 100hz [37] indicate that the retina is not rate limiting for luminance flicker fusion. Also cells in magnocellular brain areas, which are specialized for processing specific motion-directions, respond to stimuli of high temporal frequencies [25], [26]. In addition, lesion studies in non-human primates indicate that the magnocellular visual pathway [19], [20] and occipital lobe processing [6], [21] are required to detect relatively high-frequency flickering stimuli.

What is the underlying mechanism of the plasticity that results in this learning? We suggest that sensory plasticity occurs through a reinforcement-learning signal. This reinforcement signal is likely mediated by neurotransmitters such as acetylcholine, noradrenalin and dopamine, which are widely released from subcortical brain areas in a task-specific manner [38], [39] and have been implicated in neuronal plasticity [40]–[42]. Specifically, we propose that when subjects detect the targets of the letter task this reinforcement signal results in plasticity of neurons that are active at that time. Visual neurons, which are responsive to the weak visual motion-direction signals, may thus increase their responses to this visual stimulus via the learning signal and in doing so improve sensitivity to that motion-direction.

We believe that improvement in CFFT occurs because the same cells that underlie the perception of motion-directionality may also underlie the perception of flicker. Given this, if the inputs to a population of cells involved in motion-direction discrimination are strengthened, then presumably perceptions based upon those same inputs to, but perhaps different outputs from, those cells would also be affected. A likely locus of plasticity would be cells in the middle temporal visual area (MT), which have responses strongly correlated with psychophysical thresholds for coherent motion-directions [43] and have been shown to underlie perceptual learning of motion-direction discrimination [44]. Cells in MT are known to respond to low contrast motion displays [45], similar to those used in this experiment, and respond to high temporal frequencies [46].

The fact that we found an improvement in CFFT associated with improvements in motion-discrimination specific to a particular direction of motion in no way implies that CFFT improvements require direction specific learning. In fact, subjects in the CFFT Improving Group showed improvements in motion-discrimination that generalized across directions. The reason that the direction specific improvement found for the Direction-Training Group is relevant is that the direction-specific effects rule out the possibility that the improvement in CFFT was a result of a general improvement for all visual abilities in subjects. Presumably, greater improvements in CFFT would be found for training procedures that resulted in improvements in motion-direction-sensitivity that spanned multiple motion directions.

While the training procedure used in these studies is rather specialized and run in a laboratory setting, the conditions of training are similar to video games, where game-targets are often associated with moving stimuli, and which have been shown to result in perceptual learning [47]. While it remains to be empirically determined if similar elevations of CFFT will be found in other tasks, it is very possible that such CFFT elevations are common in a society that has an ever increasing reliance on video devices for work and entertainment.

While CFFT may be unreliable as a general measure of cortical processing it is likely to be useful in patient populations suffering from deficits that are sensory in nature. For instance patients with ocular disease [14], [48], and certain parietal [49] and occipital lesions [50] have reduced CFFT. In addition, patients with language disabilities such as dyslexia have reduced CFFT [51]. Our training procedure may have therapeutic value for these patients. Other researchers have found that improvements of temporal and motion processing abilities, show benefits that transfer to language abilities [52], [53]. Our training procedure may be helpful in rehabilitative settings since the stimuli that are learned (i.e. CFFT and motion direction) in our procedure are different from those on which that task is performed (i.e. letter identification). In this manner patients could be trained on a task in which they are not impoverished and gain benefits specific to their particular sensory deficit. While such possibilities are exciting to contemplate further research will be required to test these ideas and devise appropriate procedures.

## Methods

### Participants

Twenty-six participants (age 19–35 years) were recruited from the Phoenix metropolitan area. The subjects were paid the sum of $100 each for participating in the study. They attended an one and one-half hour session for 15 of 21 days (no testing occurred during the weekends). The 15 research days consisted of a three-day pre-test phase in which a total of seven tests were administered, followed by a nine-day training stage, and ultimately, a three-day post-test phase in which the initial seven tests were re-administered (data from a subset of tests are reported here). All subjects reported good ocular health and had a best-corrected visual acuity (tested on-site) of 20/40 or better (Snellen). Additionally, all participants were naive as to the purpose of the study. Informed consent was obtained from all participants, and this study conformed to the tenants of the Declaration of Helsinki for the ethical treatment of human subjects.

### Motion Stimuli

Stimuli were presented on 19” CRT monitors at a resolution of 1280×768 at 75hz controlled by Macintosh G4 Computers running OS 9.2.2. Experiments were run using custom software. Subjects viewed the display at a distance of 3 ft. and their head movements were constrained with a chinrest. Motion stimuli consisted of 200 white dots (0.2 degree radius) on a black background in a 1°–10° annulus with a dot density of 16.7 dots per deg^2^ and dot speed of 12 deg/s. Each dot had a 3-frame lifetime. At each frame-transition, a new subset of dots was chosen to move in the coherent direction while the rest of dots moved in random directions. RMS Contrast for the motion stimuli was calculated as the standard deviation of the mean luminance of the stimulus [54], [55]: (sum [p(i) *(L(i) - Lm)^2^])^1/2^ where p(i) is the proportion of pixels with luminance L(i), and Lm is the mean luminance of the stimulus. Lm is sum [p(i)* L(i)].

### Main Condition

The task-irrelevant perceptual learning paradigm [27], [29] was used for this study (see Figure 1). The experiment consisted of three phases. First, in a pre-test, each subject's performance on low luminance contrast and low motion coherence displays was evaluated. In the training phase, subjects completed nine sessions of the letter-pairing task. Finally, in the post-test, each subject's performance was re-evaluated with identical tests as used in the pre-test phase. At the beginning of each training day subjects' CFFT was evaluated (as described below).

### Motion Sensitivity Tests

For testing sessions, subjects' performance on 4 off-cardinal directions (70°, 160°, 250°, 340°) of motion was evaluated. For each trial, in all tests, a white fixation point appeared for 300 ms, and then a motion stimulus was presented for 500 ms. Subjects were then cued with a response screen to report their answer. The order of tests within each testing phase was randomized across subjects. In each trial, subjects were presented with 100% coherence motion at ten, randomly interleaved, contrasts (0, 0.14, 0.2, 0.28, 0.42, 0.6, 0.9, 1, 1.9, 11.8 cd/m^2^ RMS contrast) and asked to choose, with a mouse-click, 1 of 4 arrows that corresponded to the direction of the motion stimulus. Each direction was presented 30 times at each contrast level, thus subjects completed 1200 trials each session.

### Training Sessions

During each of the nine days of the training stage, subjects performed a rapid serial visual presentation (RSVP) letter-identification task. A sequence of 8 letters was presented in a central (1 degree) circle, after which the subject reported the two target-letters. Target-letters were either light-letters in a series of dark-distractors, or dark-letters in a series of light-distractors. Letter presentation was 375 ms temporally centered in a 500 ms motion presentation. Light-letters were 5% contrast and dark-letters were −5% contrast. While the subject performed the RSVP task, 100% coherent motion stimuli were presented in a peripheral annulus (1–10 degree). One motion direction temporally overlapped each target letter (paired-direction), and other directions temporally overlapped the distractors (non-paired directions). The paired-direction was randomly chosen, from the testing set, for each subject. The motion-stimuli were presented at 0.14 cd/m^2^ RMS contrast; at this contrast level subjects showed chance performance in the motion-direction sensitivity tests.

### CFFT Measurements

A Macular Pigment Densitometer [56] was used to measure critical flicker fusion thresholds (CFFT). CFFT was calculated psychophysically by measuring each subject's sensitivity to light flickered between blue (peak wavelength = 460 nm at 4.3 cd/m^2^) and green (peak wavelength = 550 nm at 1.5 cd/m^2^) in a 1° circle on a black background. These lights were not equiluminant and thus the percept primarily consisted of luminance flicker (i.e. subjects perceived a flickering blue light). The room was dimly lit (1.5 cd/m^2^), and lighting conditions were constant across sessions.

The method of limits was used to determine threshold values. Stimuli were presented in Maxwellian View, and participants used a chin rest throughout this part of the study. CFF was presented as a uniform spot consisting of one degree of visual angle focused in a circular region surrounding the fovea. Flicker was measured through equal counter-phased modulations of the blue light source, with the green light being fixed. The experimenter adjusted the rate of modulation, and the participant was unable to see either the control box or the researcher's actions. CFFT was defined as the mean between the frequency (Hz) at which the participant could no longer detect flicker in the stimulus and the frequency at which the participant reported that the flicker recommenced.

Subjects were divided into two experimental groups. The five subjects in the Direction-Training Group had their CFFT measured every day during the pre-test and post-test phases, as well as before each of the nine training sessions. Meanwhile, the three members of the PreTest-PostTest Group had their CFFT measured only for the pre and post tests. Additionally, two control groups (CFFT-Only) of four and five subjects, respectively, had their CFFT measured in a fashion similar to that of the experimental groups, but these subjects did not conduct the training sessions.

### Control Conditions

Four additional control experiments were run with identical methods as used for the Direction-Training Group with the exception as stated here (see Figure 1). For all subjects, CFFT was measured every day during the nine-day training stage.

#### No-Motion Group

The five subjects in the No-Motion Group were trained with the RSVP task without the dot-motion background; this was accomplished by setting the luminance of the dots to the same value as that of the background.

#### No-Coherence Group

The five subjects in the No-Coherence Group conducted the RSVP task with the background motion at 0% coherence.

#### N-Back Groups

Subjects in the N-Back and N-Back-0% Groups conducted an n-back task instead of the standard RSVP task during training. In the n-back task, the actual stimuli presented were identical to those of the RSVP task, but subjects were ask to report whether any letter was presented twice in a given trial (8 characters). If a letter was repeated, then subjects responded by pressing that letter twice (on the keyboard), or, if no letters repeated, strike the space bar twice. Subjects in the N-Back Group conducted the n-back task with a 100% coherent dot-motion background and subjects in N-Back-0% Group conducted the same task but with a 0% coherent dot-motion background.

## References

[1] Plateau J (1829). On certain properties of the impressions produced by light upon the organ of sight..

[2] Lee BB, Pokorny J, Smith VC, Martin PR, Valberg A (1990). Luminance and chromatic modulation sensitivity of macaque ganglion cells and human observers.. J Opt Soc Am A.

[3] Derrington AM, Lennie P (1984). Spatial and temporal contrast sensitivities of neurones in lateral geniculate nucleus of macaque.. J Physiol.

[4] Zlody RL (1965). The relationship between critical flicker frequency (CFF) and several intellectual measures.. Am J Psychol.

[5] Tanner WP (1950). A Preliminary Investigation of the Relationship between Visual Function of Intermittent Light and Intelligence.. Science.

[6] Halstead WC (1947). Brain and intelligence; a quantitative study of the frontal lobes..

[7] Parkin C, Kerr JS, Hindmarch I (1997). The effects of practice on choice reaction time and critical flicker fusion thresholds.. Human Psychopharmacology.

[8] McClelland G (1987). The effects of practice on measures of performance.. Human Psychopharmacology.

[9] Misiak H (1948). Practice Effect on Critical Flicker Frequency Measures.. The Journal of General Psychology.

[10] Saucer TS, Sweetbaum H (1958). Perception of the Shortest Noticebale Dark Time be Schizophrenics.. Science.

[11] Curran S, Wattis J (2000). Critical flicker fusion threshold: a potentially useful measure for the early detection of Alzheimer's disease.. Hum Psychopharmacol.

[12] Sandry M (1963). Critical flicker frequency in multiple sclerosis.. Percept Mot Skills.

[13] del Romo GB, Douthwaite WA, Elliott DB (2005). Critical flicker frequency as a potential vision technique in the presence of cataracts.. Invest Ophthalmol Vis Sci.

[14] Rota-Bartelink A (1999). The diagnostic value of automated flicker threshold perimetry.. Curr Opin Ophthalmol.

[15] Hindmarch I (1982). Critical flicker fusion frequency(cfff): the effects of psychotropic compounds.. Pharmacopsychiatria.

[16] Hindmarch I (1988). Information processing, critical flicker fusion threshold and benzodiazepines: results and speculations.. Psychopharmacol Ser.

[17] Turner P (1968). Critical flicker frequency and centrally-acting drugs.. Br J Ophthalmol.

[18] Hosokawa T, Mikami K, Saito K (1997). Basic study of the portable fatigue meter: effects of illumination, distance from eyes and age.. Ergonomics.

[19] Merigan WH, Byrne CE, Maunsell JH (1991). Does primate motion perception depend on the magnocellular pathway?. J Neurosci.

[20] Schiller PH, Logothetis NK, Charles ER (1991). Parallel pathways in the visual system: their role in perception at isoluminance.. Neuropsychologia.

[21] Mishkin M, Weiskrantz L (1959). Effects of cortical lesions in monkeys on critical flicker frequency.. J Comp Physiol Psychol.

[22] Zubek JP, Bross M (1973). Changes in critical flicker frequency during and after fourteen days of monocular deprivation.. Nature.

[23] Zubek JP, Bross M (1972). Depression and later enhancement of the critical flicker frequency during prolonged monocular deprivation.. Science.

[24] Seitz AR, Nanez JE, Holloway SR, Watanabe T (2005). Visual experience can substantially alter critical flicker fusion thresholds.. Hum Psychopharmacol.

[25] Colby CL, Duhamel JR, Goldberg ME (1993). Ventral intraparietal area of the macaque: anatomic location and visual response properties.. J Neurophysiol.

[26] Lisberger SG, Movshon JA (1999). Visual motion analysis for pursuit eye movements in area MT of macaque monkeys.. J Neurosci.

[27] Seitz AR, Watanabe T (2003). Psychophysics: Is subliminal learning really passive?. Nature.

[28] Watanabe T, Nanez JE, Koyama S, Mukai I, Liederman J (2002). Greater plasticity in lower-level than higher-level visual motion processing in a passive perceptual learning task.. Nat Neurosci.

[29] Seitz AR, Nanez JE, Holloway SR, Koyama S, Watanabe T (2005). Seeing what is not there shows the costs of perceptual learning.. Proc Natl Acad Sci U S A.

[30] Seitz A, Watanabe T (2005). A unified model for perceptual learning.. Trends Cogn Sci.

[31] Rovamo J, Raninen A (1984). Critical flicker frequency and M-scaling of stimulus size and retinal illuminance.. Vision Res.

[32] Anderson AJ, Vingrys AJ (2002). Effect of eccentricity on luminance-pedestal flicker thresholds.. Vision Res.

[33] Cavanagh P, MacLeod DI, Anstis SM (1987). Equiluminance: spatial and temporal factors and the contribution of blue-sensitive cones.. J Opt Soc Am A.

[34] Lee BB, Martin PR, Valberg A (1988). The physiological basis of heterochromatic flicker photometry demonstrated in the ganglion cells of the macaque retina.. J Physiol.

[35] Harvey LO (1970). Critical flicker frequency as a function of viewing distance, stimulus size and luminance.. Vision Res.

[36] Ernst W (1968). The dependence of critical flicker frequency and the rod threshold on the state of adaptation of the eye.. Vision Res.

[37] Williams PE, Mechler F, Gordon J, Shapley R, Hawken MJ (2004). Entrainment to video displays in primary visual cortex of macaque and humans.. J Neurosci.

[38] Schultz W (2000). Multiple reward signals in the brain.. Nat Rev Neurosci.

[39] Dalley JW, McGaughy J, O'Connell MT, Cardinal RN, Levita L (2001). Distinct changes in cortical acetylcholine and noradrenaline efflux during contingent and noncontingent performance of a visual attentional task.. J Neurosci.

[40] Arnsten AF (1997). Catecholamine regulation of the prefrontal cortex.. J Psychopharmacol.

[41] Bao S, Chan VT, Merzenich MM (2001). Cortical remodelling induced by activity of ventral tegmental dopamine neurons.. Nature.

[42] Kilgard MP, Merzenich MM (1998). Cortical map reorganization enabled by nucleus basalis activity.. Science.

[43] Britten KH, Shadlen MN, Newsome WT, Movshon JA (1992). The analysis of visual motion: a comparison of neuronal and psychophysical performance.. J Neurosci.

[44] Zohary E, Celebrini S, Britten KH, Newsome WT (1994). Neuronal plasticity that underlies improvement in perceptual performance.. Science.

[45] Sclar G, Maunsell JH, Lennie P (1990). Coding of image contrast in central visual pathways of the macaque monkey.. Vision Res.

[46] Priebe NJ, Cassanello CR, Lisberger SG (2003). The neural representation of speed in macaque area MT/V5.. J Neurosci.

[47] Green CS, Bavelier D (2003). Action video game modifies visual selective attention.. Nature.

[48] Yoshiyama KK, Johnson CA (1997). Which method of flicker perimetry is most effective for detection of glaucomatous visual field loss?. Invest Ophthalmol Vis Sci.

[49] Battelli L, Cavanagh P, Thornton IM (2003). Perception of biological motion in parietal patients.. Neuropsychologia.

[50] Vaina LM, Makris N, Kennedy D, Cowey A (1998). The selective impairment of the perception of first-order motion by unilateral cortical brain damage.. Vis Neurosci.

[51] Livingstone MS, Rosen GD, Drislane FW, Galaburda AM (1991). Physiological and anatomical evidence for a magnocellular defect in developmental dyslexia.. Proc Natl Acad Sci U S A.

[52] Merzenich MM, Jenkins WM, Johnston P, Schreiner C, Miller SL (1996). Temporal processing deficits of language-learning impaired children ameliorated by training.. Science.

[53] Tallal P, Miller SL, Bedi G, Byma G, Wang X (1996). Language comprehension in language-learning impaired children improved with acoustically modified speech.. Science.

[54] Moulden B, Kingdom F, Gatley LF (1990). The standard deviation of luminance as a metric for contrast in random-dot images.. Perception.

[55] Martinez-Trujillo J, Treue S (2002). Attentional modulation strength in cortical area MT depends on stimulus contrast.. Neuron.

[56] Wooten BR, Hammond BR, Land RI, Snodderly DM (1999). A practical method for measuring macular pigment optical density.. Invest Ophthalmol Vis Sci.

